# Effects of dietary omega-3/omega-6 fatty acid ratios on reproduction in the young breeder rooster

**DOI:** 10.1186/s12917-015-0394-9

**Published:** 2015-03-21

**Authors:** Yun Feng, Yu Ding, Juan Liu, Ye Tian, Yanzhou Yang, Shuluan Guan, Cheng Zhang

**Affiliations:** College of Life Science, Capital Normal University, Beijing, 100048 Peoples’ Republic of China; Key Laboratory of Fertility Preservation and Maintenance, Ministry of Education, Key Laboratory of Reproduction and Genetics in Ningxia, Department of Histology and Embryology, Ningxia Medical University, Ningxia, 750004 Peoples’ Republic of China

**Keywords:** PUFAs, Young breeder roosters, Reproductive hormones, Testis development, Reproductive hormone receptors, StAR mRNA

## Abstract

**Background:**

Polyunsaturated fatty acids (PUFAs) are necessary for the body's metabolism, growth and development. Although PUFAs play an important role in the regulation of reproduction, their role in testis development in the rooster is unknown. The present study was conducted to investigate the effects of omega-3/omega-6 (n-3/n-6, PUFAs) ratios on reproductive performance in young breeder roosters. Plasma levels of reproductive hormones, testis development, and reproductive hormone receptor and StAR mRNA expression were also assessed.

**Results:**

Although PUFAs (n-3/n-6: 1/4.15) had no significant effect on the testis index (P > 0.05), the spermatogonial development and germ cell layers were increased. Moreover, serum levels of hormones (GnRH, FSH, LH and T) on day 35 were also significantly increased by PUFAs (n-3/n-6: 1/4.15). To investigate whether PUFAs regulate the expression of hormone receptors and StAR, real time-PCR was used to measure GnRHR, FSHR, LHR and StAR mRNA levels. PUFAs significantly increased the mRNA levels of all of these genes.

**Conclusions:**

These results indicate that PUFAs enhance the reproductive performance of young roosters by increasing hormone secretion and function, the latter by up-regulating receptor expression. These findings provide a sound basis for a balanced n-3/n-6 PUFA ratio being beneficial to young rooster reproduction.

## Background

Reproduction is a critical part of poultry production, especially the rooster’s reproductive performance. The male gonads, testes secrete androgens and generate sperm, and are vital for males to maintain normal reproductive function. During testis development in the chicken, there is no significant increase in testicular weight from 2 to 15 weeks of age. However, the number of spermatogonia reaches more than one million [1,2]. Spermatogonia provide nutrients for sperm growth and development, and the number of spermatogonia is related to the capacity for testicular spermatogenesis. In addition, the early stage is the most important period for testicular development [3-6].

Polyunsaturated fatty acids (PUFAs) have 16 to 22 carbon atoms and more than one double bond. They have a great impact on reproduction, affecting prostaglandin (PG) synthesis, steroidogenesis, transcription factors and membrane properties [7]. Many studies showed that dietary supplementation with PUFAs, especially omega-3 (n-3) PUFAs, significantly increases sperm fertility in birds [8,9] and boars [10,11]. The long-chain n-3 PUFAs eicosapentaenoic acid (EPA; 20:5, n-3) and docosahexaenoic acid (DHA; 22:6, n-3) are especially abundant in natural sources such as fish oils and linseed oil [12]. Spermatogenesis and steroidogenesis of the avian testis depend on many gonadal hormones [13]. PUFAs such as arachidonic acid (AA) and its metabolites affect steroidogenesis through direct effects on steroid acute regulator (StAR) and cytochrome P450, which play a critical role in regulating steroid synthesis [7,14]. Meanwhile, 20-carbon PUFAs are the direct precursors of PGs [15], and participate in the regulation of reproductive endocrinology [16,17].

PUFAs influence the physical nature of cell membranes and are involved in membrane protein-mediated responses, lipid mediator generation, cell signaling and gene expression in many different cell types essential for brain and eye development and cardiac health [18]. It is reported that PUFAs may differentially affect cellular responses by changing membrane fluidity, receptor binding characteristics or their downstream activation [19-21]. Several reports suggest that the ratio of omega 6 to omega 3 fatty acids on man diet is approximately 1:1 [22,23]. Moreover, the appropriate ratio of omega6: omega3 fatty acids affect the reproductive performance of mature cockerels [8].

From 2 to 15 weeks, the growth and maturation of Sertoli and Leydig cells is an important step in early testicular development in the chicken [4]. Although n-3/n-6 (omega-3/omega-6) PUFAs are beneficial to male reproduction capacity, especially sperm quality, the appropriate ratio of n-3/n-6 PUFAs for young roosters’ testicular development and reproductive hormones was not known. The present study aimed to evaluate the effects of n-3/n-6 PUFAs ratios on testis development in young cockerels (10 to 15 weeks of age), and to investigate the possible mechanism of testis development regulation.

## Methods

### Materials

All reagents were purchased from Sigma Chemical Co. (St. Louis, MO, USA) unless otherwise specified. The ELISA kit was obtained from Nanjing Jian Cheng Bioengineering Institute (Nanjing, Jiangsu, China). Trizol reagent was obtained from Takara Bio, Takara Holdings Inc. (Otsu, Shiga, Japan). The M-MLV Reverse Transcriptase kit was purchased from Omega Bio-Tek, Inc. (Norcross, GA, USA). Soybean oil (SO) and flaxseed oil (FO) were purchased from Xin Yuan (Beijing) Fragrance Technology Co., Ltd.

### Animal treatment

Seventy-day-old Jing Hong breeder roosters (Beijing, China) were selected from the same batch and housed in cages individually under controlled environment conditions (light 13 h/dark 11 h, 22°C). The roosters were fed with dry powder feed and were given free access to feed and water. The present study was performed in accordance with the Guidelines for the Care and Use of Laboratory Animals and the China Council on Animal Care and was approved by the Institutional Animal Care and Use Committee of Capital Normal University.

The breeder roosters were randomly divided into five groups (six roosters per group at 0 days). All birds were fed diet 1 for 7 days and then received different ratios of n3/n6 PUFAs [1/18.39 (Treatment 1), 1/7.84 (Treatment 2), 1/5.04 (Treatment 3), 1/4.15 (Treatment 4) and 1/2.32 (Treatment 5)] from SO and FO. The basic formulations of the experimental diets contained n3/n6 PUFAs with different ratios from SO and FO: 2% SO/0% FO (Treatment 1), 1.5% SO/0.5% FO (Treatment 2), 1% SO/1% FO (Treatment 3), 0.5% SO/1.5% FO (Treatment 4) and 0% FO/2% SO (Treatment 5). The fatty acid compositions of the oils used in this study are presented in Table 1 and the fatty acid compositions of the diets are shown in Table 2.

**Table 1:** **Fatty acid composition of soybean oil and linseed oil (%)**

**Fatty acids**	**Soybean oil**	**Linseed oil**
C14:0	0.11	0.04
C15:0	0.02	0.02
C16:0	11.86	5.87
C16:1	0.14	0.06
C18:0	4.74	4.49
C18:1	21.60	22.30
C18:3 n-6	52.58	16.67
C18:3 n-3	7.65	49.85
C20:0	0.25	0.12
C20:1	0.19	0.16
C20:3 n-6	—	—
C20:3 n-3	—	0.06
C22:0	0.36	0.10
C22:1	0.11	0.06
C24:0	0.07	0.03
Others	0.30	0.15
Total	100.00	100.00
n-3 PUFAs	7.65	49.91
n-6 PUFAs	52.58	16.67
n-3/n-6	1:7.34	1:0.33

The values were determined by feed analysis test.

**Table 2:** **Fatty acid composition of diets (%)**

**Fatty acids**	**Treatment 1**	**Treatment 2**	**Treatment 3**	**Treatment 4**	**Treatment 5**
C14:0	0.05	0.01	0.02	0.02	0.02
C15:0	0.03	0.03	0.03	0.03	0.03
C15:1	0.02	—	—	—	—
C16:0	14.48	14.67	13.74	13.03	13.15
C16:1	0.14	0.13	0.14	0.13	0.14
C18:0	3.12	3.34	2.99	3.35	3.26
C18:1	23.58	25.63	23.91	23.29	24.13
C18:2 n-6	53.93	48.53	48.19	47.40	40.38
C18:3 n-3	2.93	6.20	9.58	11.42	17.47
C20:0	0.43	0.44	0.38	0.37	0.37
C20:1	0.28	0.29	0.28	0.31	0.32
C20:3 n-6	0.04	0.04	0.06	0.09	0.14
C20:3 n-3	—	—	—	—	—
C22:0	0.27	0.24	0.20	0.19	0.18
C23:0	0.14	0.06	0.05	0.04	0.03
C22:1	0.15	0.10	0.18	0.14	0.16
C24:0	—	—	—	—	—
Others	0.42	0.29	0.25	0.19	0.22
Total	100.00	100.00	100.00	100.00	100.00
n-3 PUFAs	53.97	48.57	48.25	47.49	40.52
n-6 PUFAs	2.93	6.20	9.58	11.42	17.47
n-3/n-6	1:18.39	1:7.84	1:5.04	1:4.15	1:2.32

The values were determined by feed analysis test.

### Collection of blood and testis samples

Blood and testis samples were collected at 21 and 35 days after treatment. Blood samples were collected by cardiac puncture [24] and were centrifuged at 3000 rpm for 10 min at 4°C to isolate plasma. Plasma was stored at −20°C until assayed for reproductive hormones.

The birds were decapitated, and their body and testis weights were measured to determine the testis index (testis weight/body weight) [1]. One of the testes was fixed in formaldehyde and embedded in paraffin. The other was stored at −80°C for RT-PCR assay.

### Histologic sections

Testes were fixed, dehydrated through a graded series of ethanol solutions and xylene, and embedded in paraffin. Testes embedded in paraffin were serially sectioned to a thickness of 3–5 μm and placed on slides coated with poly-L-lysine. The sections were stained with hematoxylin and eosin (HE) for morphological observation [1].

### Measurement of reproductive hormones

The plasma concentrations of gonadotropin-releasing hormone (GnRH), luteinizing hormone (LH), follicle-stimulating hormone (FSH) and testosterone (T) were measured using an ELISA kit (Nanjing, Jiangsu, China) according to the manufacturer’s instruction. All assays were performed in 96-well plates and the absorbance was measured at 450 nm (BioTek Instruments, Inc., Winooski, USA). A standard curve was used to determine hormone levels.

### RNA extraction, cDNA synthesis, and real-time PCR analysis

Testes were ground in liquid nitrogen, and total RNA was extracted using Trizol reagent according to the manufacturer’s instructions. Total RNA was reverse transcribed to cDNA using M-MLV Reverse Transcriptase. Briefly, 0.2 μg of total RNA was reverse transcribed in a 20-μl reaction containing 4 μl of 5× reaction buffer, 2 μl of 10 mM dNTPs, 20 U of RNase inhibitor, 200 U of RevertAid H Minus M-MULV RT enzyme, random decamer primers and RNase-free H_2_O. Quantitative PCR analysis of GnRHR, FSHR, LHR, StAR and β-actin was performed using a LightCycler 2.0 System (Roche Diagnostics).

The GnRHR primers used for amplification were a 5' forward primer (5'- ACGAGCCATGCAGCAGAAG -3') and a 3'reverse primer (5'- CGAACAGTGGAAGGAACCC -3'). The FSHR primer sequences were 5'- CATGTCTCCGGCAAAGCAA -3' (5' forward primer) and 5'- AAAACGCGTGCCATAATGG -3'(3' reverse primer). The LHR primers used for amplification were a 5' forward primer (5' - ACTCCTGCGCAAACCCATTC -3') and a 3'reverse primer (5'- CTCGGCTCTTACAGCAACCT -3'). The StAR primer sequences used were a 5'forward primer (5'- TCAGCCGGCGGATTTAAGG -3') and a 3' reverse primer (5'- TGGTGGCTGCTACAAACACT-3'). β-actin primer sequences were 5'- AACACCCACACCCCTGTGAT -3' (5' forward primer) and 5'- TGAGTCAAGCGCCAAAAGAA -3'(3' reverse primer).

The reactions were incubated in a 96-well plate at 95 °C for 5 min, followed by 40 cycles at 95 °C for 15 s and 60 °C for 1 min. Relative mRNA abundance was determined using ABI PRISM 7500 software (Applied Biosystems, Grand Island, NY, USA ). To avoid false-positive signals, dissociation-curve analyses were performed after the amplification and the PCR products were separated on a 1.5% agarose gel to confirm their sizes. Moreover, the PCR products were purified and sequenced to verify their identities. The results were normalized to the expression levels of β-actin, a housekeeping gene, by the 2-ΔΔCt method [25]. PCR reactions without reverse-transcribed cDNA were used as negative controls. The reactions were conducted in at least duplicate.

### Statistical analysis

Results are presented as means ± SEM of at least three independent experiments, as detailed in the figure legends. All data were subjected to one way (repeated-measure) ANOVA (Prism 5.0 statistical software; GraphPad Software, Inc., San Diego, CA). Significant differences between treatment groups were determined by the Tukey's test. Statistical significance was defined at P < 0.05.

## Results

### Effects of n-3/n-6 PUFAs ratios on the testis index

To evaluate the effect of n-3/n-6 PUFAs on the testis index, various ratios of n-3/n-6 PUFAs were supplied in the diet. Body weight on day 21 tended to increase with increasing ratio of n-3/n-6 PUFAs (Table 3). There were significant differences among treatment 1/2 and treatment 3/4 (P < 0.05). However, weight was not significantly increased with the highest ratio compared with the control group. Although testis weight and testis index increased in a dose-dependent manner, there were no significant differences among treatments on day 35 (P > 0.05).

**Table 3:** **Effects of different ratios of N-3/N-6 PUFAs on the testes index**

**Time**	**Treatment**	**Body weight(kg)**	**Left testis weight(g)**	**Right testis weight(g)**	**Testis weight(g)**	**Testis index**
21d	1	1.2157 ± 0.0779^a^	0.2463 ± 0.0289	0.2610 ± 0.0291	0.5740 ± 0.1245	0.4185 ± 0.0432
	2	1.3115 ± 0.0542^a^	0.2883 ± 0.0339	0.3030 ± 0.0325	0.4580 ± 0.1344	0.4483 ± 0.0315
	3	1.4752 ± 0.0139^b^	0.3117 ± 0.0314	0.3223 ± 0.0364	0.6307 ± 0.0707	0.4304 ± 0.0483
	4	1.3922 ± 0.0161^b^	0.3683 ± 0.0352	0.3807 ± 0.0349	0.9063 ± 0.1492	0.5382 ± 0.0508
	5	1.3055 ± 0.0610^a^	0.3637 ± 0.0562	0.3807 ± 0.0544	0.7443 ± 0.1107	0.5648 ± 0.0622
35d	1	1.5703 ± 0.0504	0.8743 ± 0.0239	0.8904 ± 0.0211	1.7647 ± 0.0451	1.1276 ± 0.0610
	2	1.5948 ± 0.0431	0.8890 ± 0.0191	0.9103 ± 0.0136	1.7993 ± 0.0321	1.1294 ± 0.0298
	3	1.5680 ± 0.0264	0.8882 ± 0.0078	0.9025 ± 0.0134	1.7907 ± 0.0208	1.1428 ± 0.02678
	4	1.6027 ± 0.0440	0.9050 ± 0.0128	0.9317 ± 0.0093	1.8367 ± 0.0218	1.14789 ± 0.0371
	5	1.5797 ± 0.0170	0.8923 ± 0.0482	0.9167 ± 0.0673	1.8090 ± 0.0113	1.1423 ± 0.0058

Testis index = testis weight (g)/body weight (kg).

In the same column, values with different small letter superscripts mean significant difference (P < 0.05).

### Effects of N-3/N-6 PUFAs ratios on testis morphology

**Figure 1:**
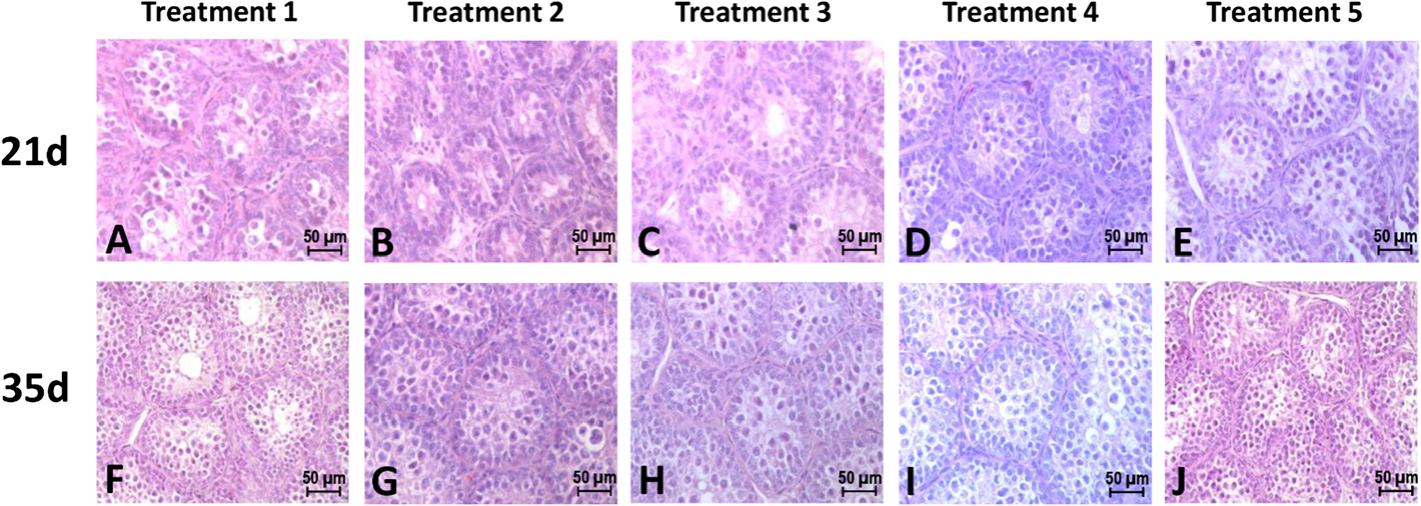
**Effects of different ratio of n-3/n-6 on morphology of chicken testis. A**, **B**, **C**, **D** and **E** were the morphology of chicken testis on day 21, and **F**, **G**, **H**, **I** and **J** were the the morphology of chicken testis on day 35 for treatment 1–5. Magnification, × 400; Bar = 50 μm.

**Figure 2:**
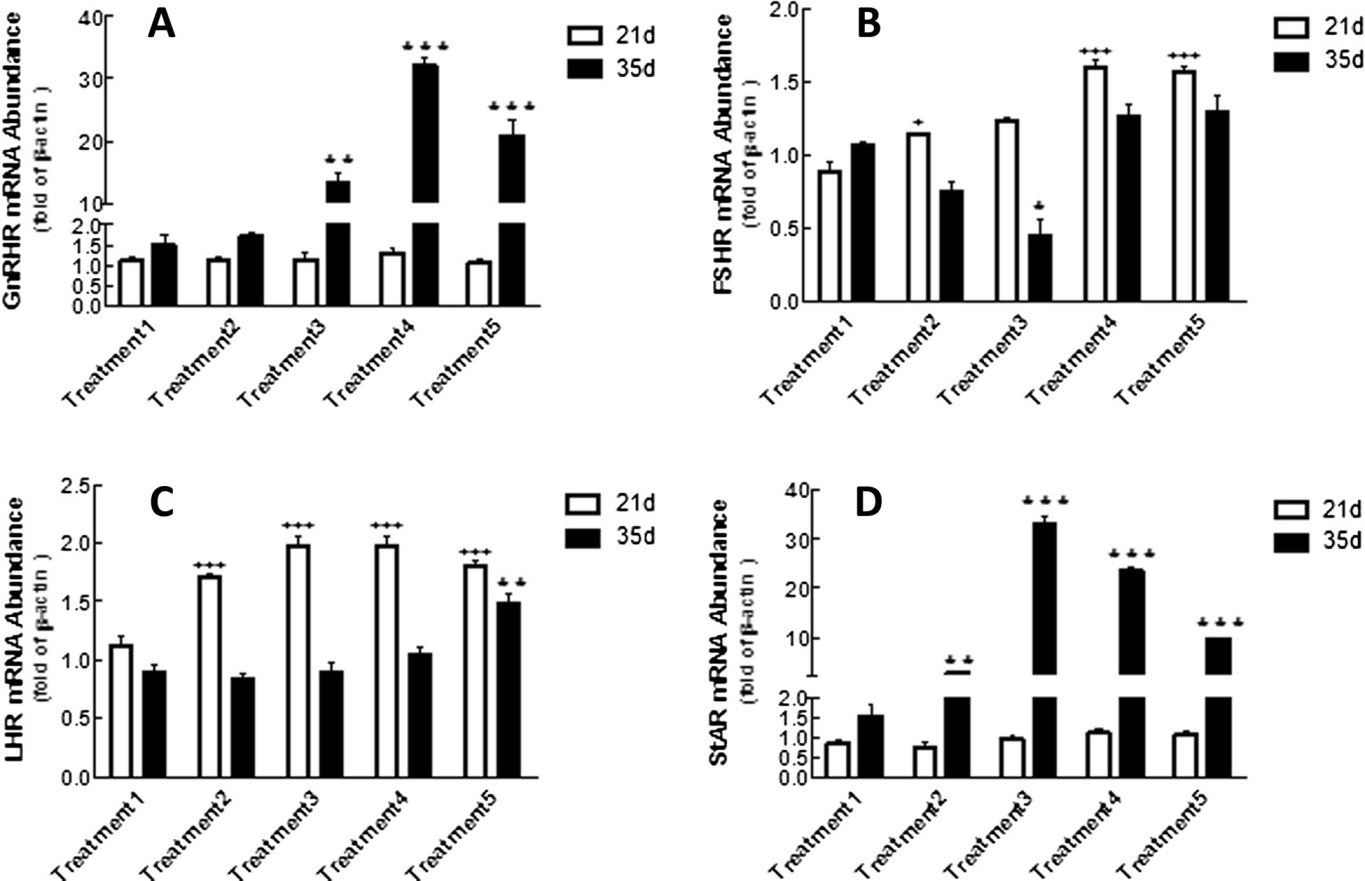
**The mRNA relative expression of GnRHR, LHR and FSHR in chicken testis.** The testis were collected for mRNA (real-time PCR) analysis. The mRNA abundance were normalized by β-actin. **(A)** Although the different treatments had no significant influence on GnRH content on day 21, the response was significantly up-regulated by the presence of n3/n6 (treatment 3, 4 and 5) on day 35 (***P < 0.001). **(B)** Treatment 2, 4 and 5 significantly increased FSHR mRNA compared with others treatments on day 21 (^+++^P < 0.001). **(C)** Diet with different ratios of n3/n6 PUFAs (treatment 2–5) significantly increased LHR mRNA level on day 21 (^+++^P < 0.001). The effect of PUFAs was decreased with the duration of treatment on day 35. **(D)** Although PUFAs had no significant effect on StAR content on day 21, the treatment 2–5 dramatically increased StAR mRNA level (**P < 0.01; ***P < 0.001). ^+^ indicates significant difference among treatments on day 21. * indicates significant difference among treatments on day 35.

Testis morphology in birds at 21 and 35 days are shown in Figure 1. The seminiferous tubule epithelium and spermatogonia of the underlying epithelial cells developed normally. Although no significant difference in testis morphology was found among treatments, 5–6 layers of germ cells were seen on day 21 in birds given treatment 4 (Figure 1D) and treatments 5 (Figure 1 E). There were 2–3 layers of germ cells with treatments 1 (Figure 1A) and 2 (Figure 1B) and 3–4 layers with treatments 3 (Figure 1C).

On day 35, 6–7 layers of germ cells were observed in the treatment 5 (Figure 1J). The formation speed of spermatogonial at day 35 was improved with an increase in the ratio of n-3/n-6 PUFAs.

### Effects of n-3/n-6 PUFAs ratios on serum hormone levels

As shown in Table 4, the concentration of GnRH on day 21 and 35 increased with increasing n-3/n-6 PUFA ratio, but was lower at an n-3/n-6 PUFA ratio of 1/2.32 (treatment 5). The GnRH concentration at 21 and 35 days were significantly higher for treatment 4 compared with the other treatments (P < 0.05). The LH and T concentrations on days 21 and 35 and the FSH concentration on day 35 showed similar trends as that for GnRH concentration: the LH, FSH and T concentrations were significantly higher for treatment 4 than for other treatments (P < 0.05). There were no significant differences in FSH concentrations at 21 days.

**Table 4:** **Effects of different ratios of N-3/N-6 PUFAs on the serum hormone levels**

**Time**	**Treatment**	**GnRH(ng/L)**	**LH(mIU/ml)**	**FSH(IU/L)**	**T(nmo/L)**
21d	1	114.8510 ± 3.6500^a^	2.1117 ± 0.0816^b^	5.3316 ± 0.0935	9.4830 ± 0.2194^a^
	2	117.8113 ± 1.9410^a^	2.0503 ± 0.0245^ab^	5.3893 ± 0.1400	9.5000 ± 0.1830^ab^
	3	117.9606 ± 4.5833^a^	2.1187 ± 0.2904^b^	5.2857 ± 0.1560	9.5286 ± 0.5078^ab^
	4	128.3990 ± 4.2219^b^	2.3397 ± 0.1090^b^	5.5743 ± 0.2446	10.6810 ± 0.3215^b^
	5	118.7643 ± 5.0145^a^	1.6170 ± 0.0758^a^	5.3740 ± 0.1878	9.4426 ± 0.1897^ab^
35d	1	118.0626 ± 5.9451^a^	2.2897 ± 0.1448^a^	3.8223 ± 0.0789^a^	20.9670 ± 0.2361^a^
	2	124.7476 ± 1.7489^a^	2.8570 ± 0.1251^a^	4.2557 ± 0.1354^a^	20.8390 ± 0.4256^a^
	3	120.1923 ± 3.4892^a^	2.7343 ± 0.1044^a^	3.7997 ± 0.0837^a^	21.3133 ± 0.8362^ab^
	4	136.6667 ± 3.0792^b^	3.4170 ± 0.1306^b^	4.6900 ± 0.1718^b^	23.6027 ± 0.3857^b^
	5	122.5713 ± 2.0841^a^	2.7203 ± 0.1524^a^	4.3910 ± 0.3484^a^	20.2320 ± 1.3243^a^

In the same column, values with different small letter superscripts mean significant difference (P < 0.05).

### Effects of n-3/n-6 PUFAs ratios on the mRNA levels of hormone receptor genes in the testis of breeder roosters

The relative mRNA levels of GnRH receptor (GnRHR), FSH receptor (FSHR) and LH receptor (LHR) in the chicken testis are shown in Figure 2. There were no differences among treatments in GnRHR mRNA expression on day 21 (Figure 2A). However, the mRNA level of GnRH was significantly increased by n3/n6 PUFAs, especially an n3/n6 ratio of 1/4.15 (Figure 2A).

FSHR expression on day 21 was significantly increased in a dose-dependent manner (P < 0.001) (Figure 2B) and was higher for treatment 4 compared with other treatments. Although treatment 3 significantly decreased FSHR expression on day 35 (P < 0.01), there were no differences among treatments 1, 2, 4 and 5 (P > 0.05) (Figure 2B).

The LHR mRNA level was lower for treatment 1 compared with the other treatments on day 21 (P < 0.001) (Figure 2C), and was highest for treatment 5 on day 35 (P < 0.01) (Figure 2C). There were no differences among treatments 1, 2, 3 and 4 (P > 0.05).

### Effects of n-3/n-6 PUFAs ratios on the mRNA level of the StAR gene in the testis of breeder roosters

As show in Figure 2D, StAR mRNA expression in the rooster testis on day 21 was not significantly affected by the n-3/n-6 ratio, although treatments 4 and 5 increased the StAR mRNA level compared with treatment 1 (P > 0.05) (Figure 2D). At 35 days, treatments 3, 4 and 5 showed significant increases in StAR mRNA level (P < 0.001) (Figure 2D).

## Discussion

The present study aimed to evaluate the effect of different n-3/n-6 PUFAs ratios on testicular development and reproductive hormones, and to provide the basis for determining the appropriate n-3/n-6 PUFA ratio for young chickens. Previous research on boars [10,11], rats [26] and birds [8,9] showed that consumption of an appropriate ratio of n-3/n-6 PUFAs was beneficial to male reproduction capacity, especially sperm quality. However, the appropriate ratio of n-3/n-6 PUFAs for testicular development and reproductive hormones in young roosters was not known.

Testes of chickens are located in the dorsal abdomen and are oval- or bean-shaped and milky white. Early testicular development is crucial to improving the reproductive performance of roosters. From 2 to 15 weeks, the growth and maturation of Sertoli and Leydig cells is the important step in early testicular development in the chicken [4]. These cells facilitate the development of pluripotent PGCs into spermatogonia, which support the germinal cells throughout the life of the bird [27]. The period from 10 to 15 weeks is the key stage for later testicular development. Many studies have shown that testicular growth and development are delayed in underweight chicken [28,29]. The ratio of testis weight to total body weight can reflect testis growth. Yan et al. [26] found that a variety of ratios of n-3/n-6 PUFAs had no effect on the testis index in Sprague–Dawley rats. Our study had a similar result for the testis index, although the testis index tended to increase with increasing n-3/n-6 PUFA ratio.

Both n-6 and n-3 PUFAs affect reproduction. Many studies have shown the effects of n-3/n-6 PUFA ratio on male reproduction. GnRH, which is released by the hypothalamus, stimulates release of FSH and LH from the pituitary gland [30]. It is reported that a high-energy diet increased GnRH pulse frequency, testicular weight and sperm production [31]. The study indicated that spermatogenesis and steroidogenesis in the avian testis are dependent on FSH, LH and T [6]. FSH binds to its receptors in the membranes of Sertoli cells, and stimulates spermatogenesis. Testicular function is associated with FSH concentrations in male broiler breeders, and that testis weight is highly correlated with FSH [6]. On the other hand, FSH is necessary for initiation of spermatogenesis and maturation of spermatozoa. Meanwhile, the numbers of spermatogonia, spermatocytes and sperm cells would increase in male monkeys given recombinant human FSH [32]. FSH and LH regulate spermatogenesis via cyclic adenosine 3’, 5’-monophosphate (cAMP) [33]. LH binds to receptors in the membranes of Leydig cells, and stimulates the secretion of T. T levels determine the testicular development and behavior of roosters [26,31]. T may act on the Sertoli and peritubular cells of the seminiferous tubules and stimulate spermatogenesis [34]. In the present study, we showed that the concentrations of GnRH, FSH, LH and T were positively related to the quality and morphology of sperm. Similarly, Yan et al. [26] reported that the concentrations of GnRH, FSH, LH and T increased with increasing n-3/n-6 PUFA ratio, and that lower and higher n-3/n-6 ratios have opposite effects on reproduction. These results indicate that an appropriate n-3/n-6 PUFA ratio is important for the development of spermatogonia in the rooster. From morphologic analyses of testes, treatments 4 and 5 yielded better histological changes. This suggests that the increased hormone levels improved testis development.

PUFAs act via cell surface and intracellular receptors/sensors that control cell signaling and gene expression patterns [35]. Some effects of n-3 PUFAs appear to be mediated by, or at least associated with, changes in the fatty acid composition of cell membranes. GnRHR, FSHR and LHR have important roles in the regulation of male reproduction [16,36,37]. In the present study, relative GnRHR, FSHR and LHR mRNA levels at 21 and 35 days differed significantly among ratios of n-3/n-6 PUFAs. This evidence suggests that PUFAs may affect cellular responses through changes in membrane fluidity, receptor binding characteristics or their downstream activation.

StAR promoter activity and StAR mRNA and protein levels in MA-10 Leydig cells were inhibited by inhibition of endogenous AA release, whereas addition of exogenous AA reversed these effects [16]. On the other hand, specific inhibition of PTGS2 was also associated with increased StAR expression [17]. We found that the relative StAR mRNA level on day 35 differed significantly among ratios of n-3/n-6 PUFAs.

## Conclusions

In conclusion, dietary treatment of roosters with an appropriate n-3/n-6 PUFA ratio (treatment 4: 1/4.15) increased hormone secretion, thereby improving testis development. The treatment also increased reproductive performance, which may be related to changes in the mRNA levels of hormone receptors and StAR. These findings provide a sound basis for a balanced n-3/n-6 PUFA ratio being beneficial to young rooster reproduction.

## Abbreviations

PUFAs: Polyunsaturated fatty acids
DHA: Docosahexaenoic
SO: Soybean oil
LO: Linseed oil
GnRH: Gonadotropin releasing hormone
FSH: Follicular stimulating hormone
LH: Luteinizing hormone
T: Testosterone
StAR: Steroid acute regulator protein
